# Effects of rasagiline, its metabolite aminoindan and selegiline on glutamate receptor mediated signalling in the rat hippocampus slice in vitro

**DOI:** 10.1186/1471-2210-11-2

**Published:** 2011-02-21

**Authors:** W Dimpfel, JA Hoffmann

**Affiliations:** 1 Justus Liebig University Giessen, c/o NeuroCode AG, Sportparkstr. 9, D 35578 Wetzlar, Germany; 2 TEVA Pharma GmbH, Waldecker Str. 7, D 64546 Moerfelden-Walldorf, Germany

## Abstract

**Background:**

Rasagiline, a new drug developed to treat Parkinson's disease, is known to inhibit monoamine oxidase B. However, its metabolite R-(-)-aminoindan does not show this kind of activity. The present series of in vitro experiments using the rat hippocampal slice preparation deals with effects of both compounds on the pyramidal cell response after electric stimulation of the Schaffer Collaterals in comparison to selegiline, another MAO B inhibitor.

**Method:**

Stimulation of the Schaffer Collaterals by single stimuli (SS) or theta burst stimulation (TBS) resulted in stable responses of pyramidal cells measured as population spike amplitude (about 1 mV under control SS conditions or about 2 mV after TBS).

**Results:**

During the first series, this response was attenuated in the presence of rasagiline and aminoindan-to a lesser degree of selegiline-in a concentration dependent manner (5-50 μM) after single stimuli as well as under TBS. During oxygen/glucose deprivation for 10 min the amplitude of the population spike breaks down by 75%. The presence of rasagiline and aminoindan, but rarely the presence of selegiline, prevented this break down. Following glutamate receptor mediated enhancements of neuronal transmission in a second series of experiments very clear differences could be observed in comparison to the action of selegiline: NMDA receptor, AMPA receptor as well as metabotropic glutamate receptor mediated increases of transmission were concentration dependently (0,3 - 2 μM) antagonized by rasagiline and aminoindan, but not by selegiline. On the opposite, only selegiline attenuated kainate receptor mediated increases of excitability. Thus, both monoamino oxidase (MAO) B inhibitors show attenuation of glutamatergic transmission in the hippocampus but interfere with different receptor mediated excitatory modulations at low concentrations.

**Conclusions:**

Since aminoindan does not induce MAO B inhibition, these effects must be regarded as being independent from MAO B inhibition. The results provide strong evidence for a neuroprotective activity of rasagiline and aminoindan in concert with an extended clinical indication into the direction of other diseases like Alzheimer's disease or stroke.

## Background

Rasagiline (N-propargyl-1-(R)-aminoindan) and selegiline are drugs prescribed for the treatment of Parkinson's disease. Both are believed to act by inhibition of monoamine oxidase B (MAO B). However, both are metabolized in a different way: rasagiline gives rise to aminoindan, a compound reported to have neuroprotective capabilities of its own, whereas selegiline gives rise to the neurotoxic metabolite methamphetamine [1,2]. Similar electropharmacograms obtained by quantitative brain field potential analysis were obtained from freely moving rats in the presence of rasagiline and its metabolite aminoindan (not inhibiting monoamine oxidase B). Selegiline-on the other hand-produced a time dependent biphasic action presumably due to the action of its active metabolites [3]. Available evidence suggests an additional mechanism of action for these drugs independently from MAO B inhibition.

For example, a neuroprotective action unrelated to MAO inhibition has been reported by [4] for rasagiline as well as for its major metabolite 1-(R)-aminoindan [5]. For review of neuroprotective effects of rasagiline and aminoindan see [6]. But again, no final mechanism has been reported to explain the proposed neuroprotective action. There is solid evidence of an involvement of glutamatergic transmission in neuroprotection. This calls for an experimental setup to dissect the possible interference of these compounds within the glutamatergic system. To our knowledge, no neurophysiological techniques have been applied up to now to characterize the effects of these compounds on glutamatergic transmission in the hippocampus. This model should be suitable since the communication between Schaffer-Collaterals and the hippocampal pyramidal cells takes place by using glutamate as transmitter.

The hippocampus slice preparation is a validated model for direct analysis of interaction of substances with living neuronal tissue [7,8]. Due to the preservation of the three dimensional structure of the hippocampus, drug effects on the excitability of pyramidal cells can be studied in a unique manner. Electric stimulation of Schaffer Collaterals leads to release of glutamate resulting in excitation of the postsynaptic pyramidal cells. The result of the electrical stimulation can be recorded as a so-called population spike (pop-spike). The amplitude of the resulting population spike represents the number of recruited pyramidal cells and relates to the extent of glutamatergic transmission. The advantage of the model not only consists in the possibility of physiological recording in vitro during 8 hours but also to modify the excitability of the system in order to create pathophysiological conditions like transient oxygen and glucose deprivation (OGD) [9].

The first part of the present investigation aimed at the characterization of the effects of rasagiline and its metabolite aminoindan in comparison to selegiline on glutamatergic transmission within a physiological environment and under pathophysiological conditions. The principle of the second part of the investigation was to use the enhancement of the pyramidal cell response (increased amplitudes of population spike) in the presence of highly specific and selective agonists of different glutamate receptors as a challenge. Accordingly, these responses were followed in the presence of several concentrations of rasagiline, aminoindan and selegiline. This approach should reveal great similarities between rasagiline and aminoindan on one side and a great difference to the action of selegiline on the other side.

## Methods

Hippocampus slices were obtained from 43 adult male Sprague-Dawley rats (Charles River Wiga, Sulzbach, Germany). Rats were kept under a reversed day/night cycle for 2 weeks prior start of the experiments, to allow recording of in vitro activity from slices during the active phase of their circadian rhythm [10,11]. Animals were exsanguinated under ether anaesthesia, the brain was removed and the hippocampal formation was isolated under microstereoscopic sight. The midsection of the hippocampus was fixed to the table of a vibrating microtome (Rhema Labortechnik, Hofheim, Germany) using a cyanoacrylate adhesive, submerged in chilled bicarbonate-buffered saline (artificial cerebrospinal fluid (ACSF): NaCl: 124 mM, KCl: 5 mM, CaCl2: 2 mM, MgSO4: 2 mM, NaHCO3: 26 mM, glucose: 10 mM, and cut into slices of 400 μm thickness. All slices were pre-incubated for at least 1 h in Carbogen saturated ACSF (pH 7.4) in a pre-chamber before use [12].

**Figure 1:**
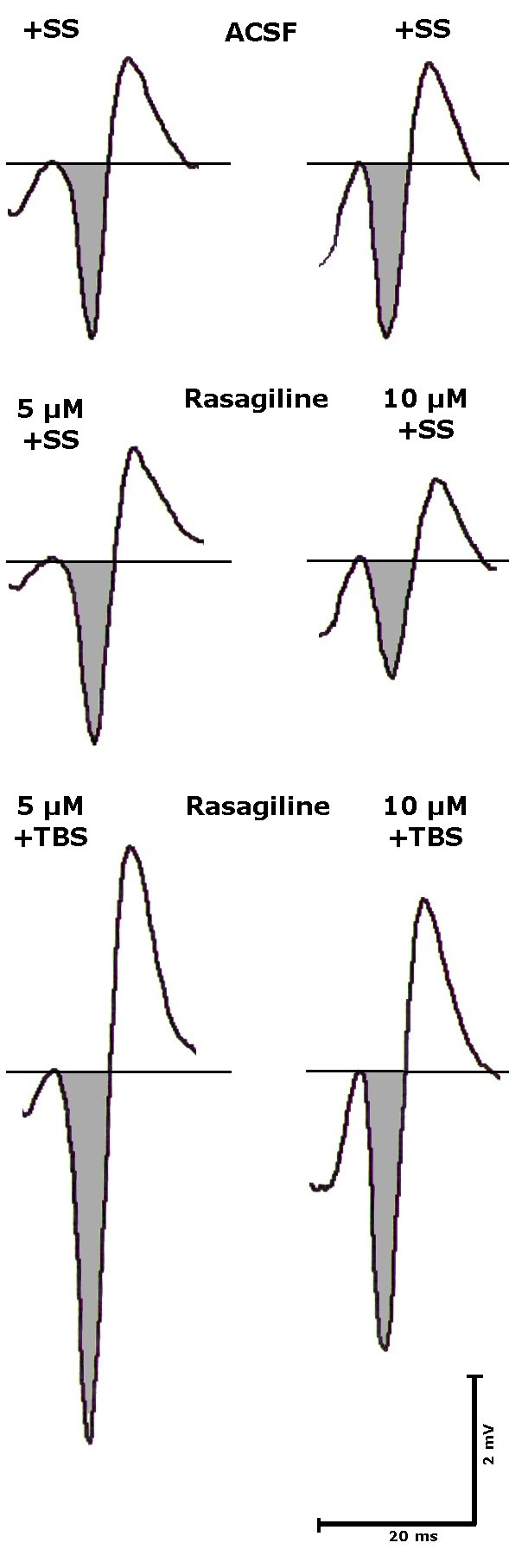
**Documentation of original signals showing the effects of using single stimuli (SS) or theta burst stimulation (TBS) in control slices (left panel) or in the presence of rasagiline (right panel) diluted in artificial cerebro-spinal fluid (ASCF)**. The amplitude is calculated from baseline to the down reflection of the signal (shadowed). Stimulus artefacts are omitted for the sake of clarity. Scales: Time is given in milliseconds (ms), amplitude in millivolt (mV).

During the experiment the slices were held and treated in a special superfusion chamber (List Electronics, Darmstadt, Germany) according to [13] at 35°C [14]. Five slices per rat were used. The preparation was superfused ACSF at 220 ml/h. Electrical stimulation (200 μA constant current pulses of 200 μs pulse width) of the Schaffer Collaterals within the CA2 area and recording of extracellular field potentials from the pyramidal cell layer of CA1 [12] was performed according to conventional electrophysiological methods using the "Labteam" Computer system "NeuroTool" software package (MediSyst GmbH, Linden, Germany). Measurements were performed at 10 min intervals in order to avoid potentiation mechanisms after single stimuli (first recording at 10 min is discarded for stability purposes). Four stimulations-each 20 s apart-were averaged for each time point. After averaging the last three of four responses to single stimuli (SS) to give one value, potentiation was induced by applying a theta burst type pattern (TBS; [7]). The mean amplitude of three signals 20 seconds apart were averaged to give the mean of absolute voltage values (microvolt) ± standard error of the mean for each experimental condition (single stimulus or theta burst stimulation). Electrical stimulation of the Schaffer Collaterals within the C2 area with single stimuli resulted in stable responses of the pyramidal cells in form of population spikes with an amplitude of about 1 mV and about 2 mV after theta burst stimulation (TBS) (representative example is given in Figure 1). Oxygen and Glucose deprivation (OGD) was performed in analogy to [15] by shutting off oxygen and glucose for 10 minutes. In this case glucose was replaced by sucrose.

For stimulation of glutamate receptors (NMDA, AMPA, Kainate and metabotropic receptor) four agonists were used, respectively: *trans*-1-Aminocyclobutan-1,3-dicarboxylic acid (ACBD; [16]), (S)-(-)-α-Amino-5-fluoro-3,4-dihydro-2,4-dioxo-1(2*H*)-pyrimidinepropanoic acid (S-Fluorowillardiine; [17-19]), (RS)-2-Amino-3-(3-hydroxy-5-tert-butyliosxazol-4-yl)propanoic acid (ATPA; [20-23]) and (±)-1-Aminocyclopentane-*trans*-1,3-dicarboxylic acid (t-ACPD; [24-26]). All agonists were tested in pilot experiments in order to detect a concentration leading to strong increases of population spike amplitude in the presence of single stimuli (SS) and theta burst stimulation (TBS). Origin of the chemicals is given in Table 1. The allowance to keep animals for this purpose was obtained from governmental authorities, dated 2009-09-01 under the document Nr. 0200052529. Experiments were performed in accordance with the German Animal Protection Law.

**Table 1:** Compounds used

rasagiline	CH.B: 255400204	TEVA Pharma GmbH
selegiline	CH.B: 0405BG/01	BIO TREND
aminoindan	CH.B:087K4619	Sigma-Aldrich Chemie GmbH
trans-ACBD	CH.B: 0048BN/01	BIO TREND
trans-ACPD	CH.B: 0053BN/01	BIO TREND
(S)-(-) S-Fluorowillardiine	CH.B: 9A/36714	BIO TREND
(RS)-ATPA	CH.B: 0096 BN/01	BIO TREND

Origin of chemical compounds used for this experimental series.

## Results

### a) Neurophysiological evidence for neuroprotective effects

**Figure 2:**
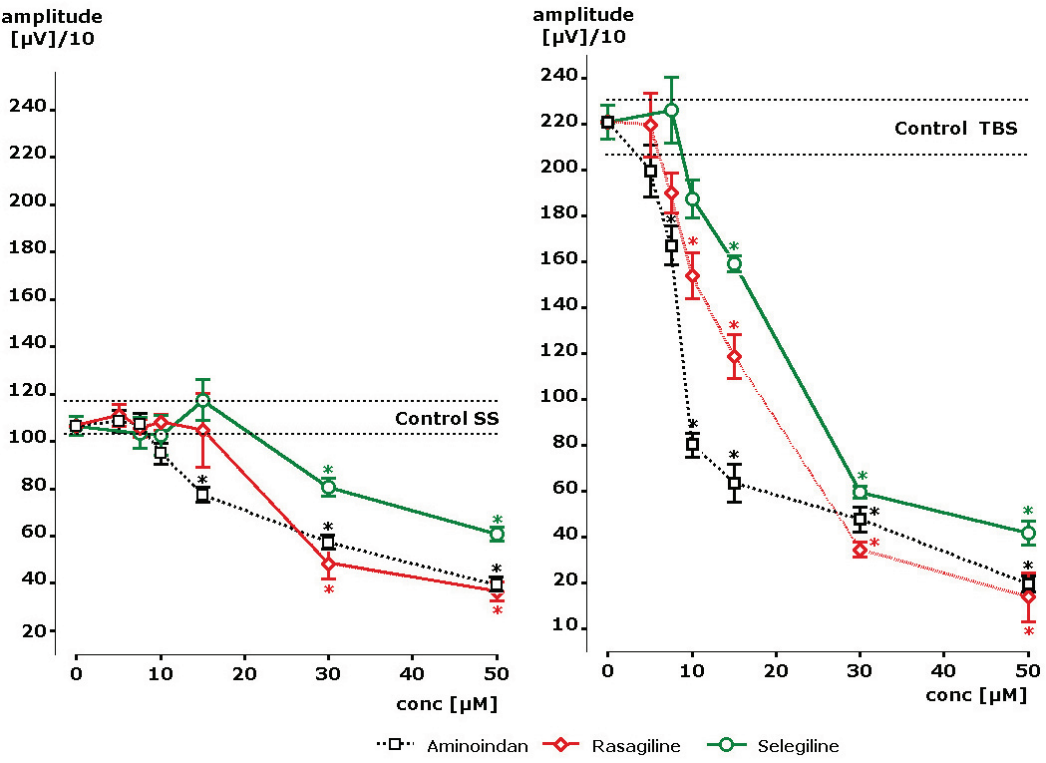
**Concentration dependent effects of rasagiline, aminoindan and selegiline on pyramidal cell activity in terms of changes of population spike amplitude**. Results from single slices as obtained after single stimuli (SS) and after theta burst stimuli (TBS). Data are given in microvolt for a mean of four slices and standard error of the mean. Stars indicate statistical significance of p < 0.05 in comparison to control.

Using single stimulus administration rasagiline and - to a lesser degree-selegiline attenuated the pyramidal cells response significantly at a concentration of 30 μM. In the presence of aminoindan, however, significant attenuation was observed already at 15 μM. At a concentration of 50 μM rasagiline and aminoindan reduced the amplitude by about 60%, selegiline by about 40%. The course of the concentration dependence is given in Figure 2 for all three compounds. Under the condition of theta burst stimuli, rasagiline was able to reduce the signal amplitude significantly at 10 μM, whereas the effect of selegiline reached statistical significance at a concentration of 15 μM. The effects of aminoindan became statistically significant already at a concentration of 7.5 μM. Thus, in the presence of rasagiline, aminoindan and selegiline a concentration dependent decrease of the amplitudes of the population spike could be observed during single shock stimulation as well as during theta burst stimulation. Effects of selegiline were weakest (Figure 2).

**Figure 3:**
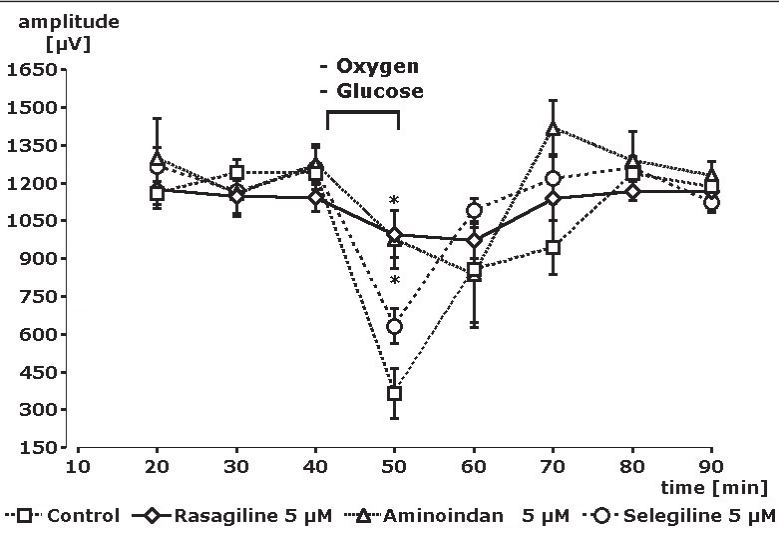
**Complete time course of experiments**. Bar indicates 10 min of oxygen and glucose deprivation (OGD) before measurement. Nearly complete prevention of OGD-induced break down of population spike amplitude (s. control) by rasagiline and aminoindan but only to a minor degree by selegiline (p < 0.1). Stars indicate statistical significance of p < 0.05 in comparison to control.

In order to proof, that this attenuation of glutamatergic transmission could be related to neuroprotective features of the compounds, a pathophysiological situation was created in slices by turning off oxygen and glucose for 10 minutes. This procedure succeeded in a breakdown of the signal amplitudes after electrical single stimuli by about 75%. This breakdown was nearly totally prevented (p < 0.05) by the presence of a concentration of 5 μM rasagiline or aminoindan in the superfusion medium but rarely by selegiline (p < 0.1). Time courses of the experiments are depicted in Figure 3. This effect was still visible but not statistically significant from control at time period 60 and 70 minutes after start of the experiment. Thus, rasagiline and aminoindan showed a clearly better neuroprotective effect than selegiline in this model.

### b) Functional interference with NMDA receptor activation

**Figure 4:**
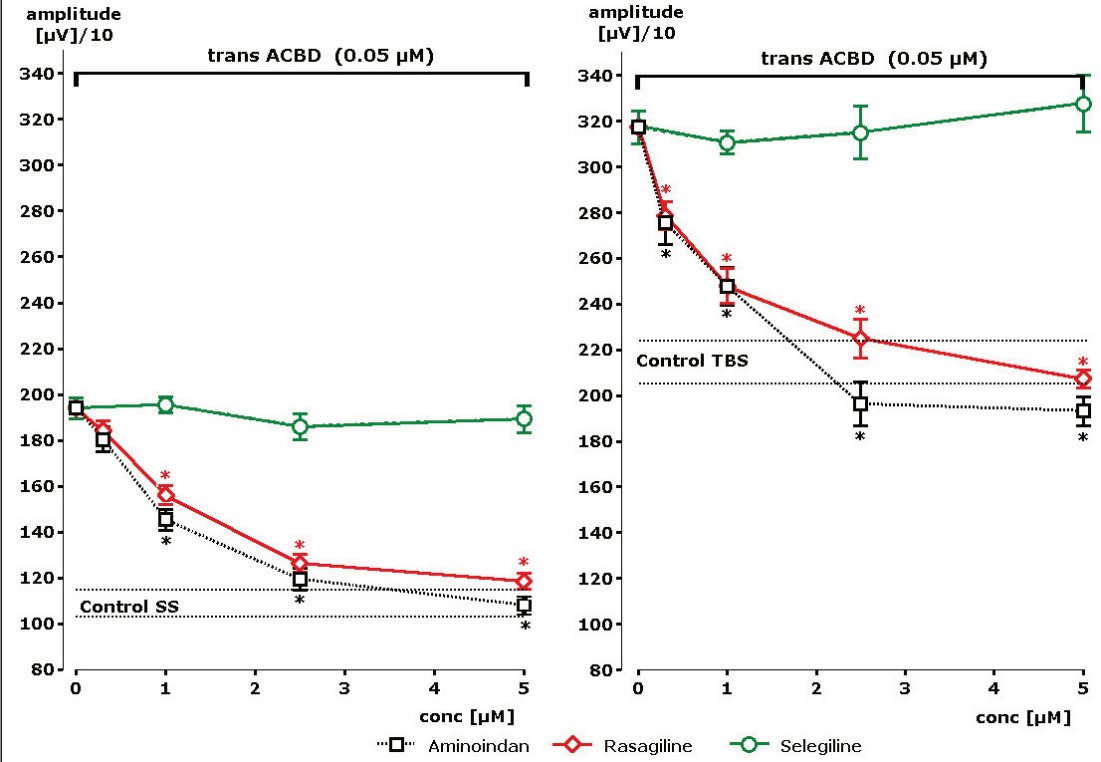
**Concentration dependent effects of rasagiline, aminoindan and selegiline in the presence of single stimuli (SS) or theta burst stimuli (TBS) after stimulation of the NMDA glutamate receptor by ACBD (n = 4 slices +- SEM)**. Statistically significant attenuation of pop-spike amplitude in comparison to ACBD-induced increases were obtained in the presence of 1 μM of rasagiline or aminoindan following single stimuli (SS). During theta burst stimulation (TBS) already a concentration of 0.3 μM of rasagiline or aminoindan became statistically significant. Stars indicate statistical significance of p < 0.05 in comparison to control.

In order to test a possible interference of rasagiline, aminoindan or selegiline with NMDA receptor activation, glutamatergic neurotransmission was modulated by ACBD, a very potent and selective NMDA receptor agonist. A concentration of 50 nM induced a significant enhancement of the population spike amplitude. Under the condition of single stimuli increase of the amplitude from 1106 to 1940 μV (176% of control value) could be observed (Figure 4). In the presence of rasagiline the amplitude remained at control value (changing from 1102 to 1185 μV). Statistically significant differences to the ACBD induced increase were already observed with a concentration of 1 μM of rasagiline (p < 0.01).

Similar results were obtained in the presence of theta burst stimulation. Presence of ACBD in the superfusion medium increased the amplitude to 3173 μV. Rasagiline at a concentration of 5 μM attenuated the ACBD-induced signal down to 2074 μV (about control value). A statistically significant difference to ACBD-induced values was obtained at the very low concentration of 300 nM of rasagiline and aminoindan (p < 0.01). Thus, a concentration dependent attenuation of NMDA receptor induced increases of population spike amplitudes was recognized. Nearly identical results were seen in the presence of aminoindan (s. Figure 4). On the opposite, virtually no effect could be seen in the presence of selegiline up to a concentration of 5 μM. Thus, a clear difference could be observed between rasagiline and aminoindan on one site and selegiline on the other side with respect to functional antagonism of NMDA glutamate receptor stimulation.

### c) Functional interference with AMPA receptor activation

**Figure 5:**
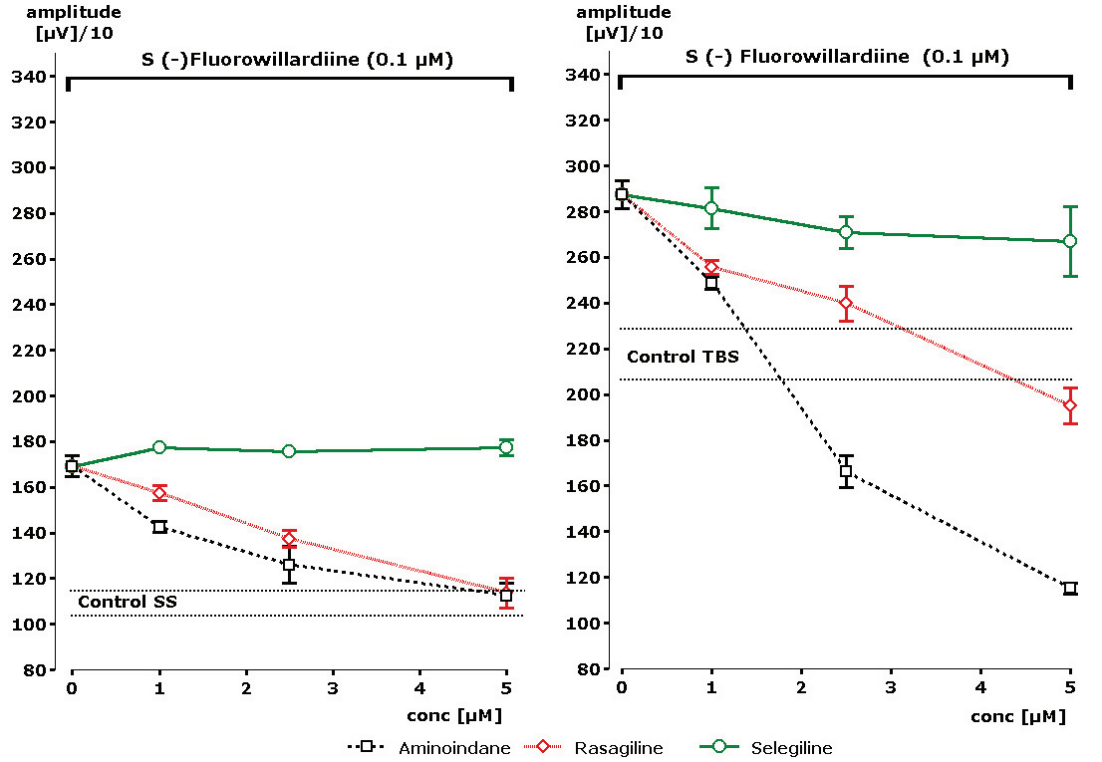
**Concentration dependent effects of rasagiline, aminoindan and selegiline in the presence of single stimuli (SS) or theta burst stimuli (TBS) after stimulation of the AMPA glutamate receptor by fluorowillardiine (n = 4 slices +- SEM)**. Statistically significant attenuation of pop-spike amplitude in comparison to fluorowillardiine-induced increases were obtained in the presence of 1 μM of rasagiline or aminoindan following single stimuli (SS). During theta burst stimulation (TBS) already a concentration of 1 μM of rasagiline or aminoindan became statistically significant. Stars indicate statistical significance of p < 0.05 in comparison to control.

In order to test a possible interference of rasagiline, aminoindan or selegiline with AMPA receptor activation, the glutamatergic neurotransmission was stimulated by fluorowillardiine, a very potent and selective AMPA receptor agonist. A concentration of 100 nM induced a significant enhancement of the population spike amplitude. Under the condition of single stimuli increase of the amplitude from 1135 to 1692 μV (151% of control value) could be observed (Figure 5). In the presence of 5 μM of rasagiline the amplitude remained at control value (changing from 1089 to 1137 μV).

Statistically significant differences to the effect of fluorowillardiine were observed with 2.5 μM of rasagiline (p < 0.02) and aminoindan (p < 0.01). Similar results were obtained in the presence of theta burst stimulation. Fluorowillardiine increased the amplitude to 2873 μV. Rasagiline at a concentration of 5 μV attenuated the fluorowillardiine-induced signal to a control value of 1950 μV. A statistically significant difference to fluorowillardiine-induced values was obtained at the very low concentration of 1 μM of rasagiline (p < 0.05).

Even stronger effects were seen in the presence of aminoindan (s. Figure 2). Aminoindan attenuated the amplitude of the population spike from 2888 μV down to 1152 μV, which is far beyond the control values. Statistical significance in comparison to AMPA receptor stimulation was obtained already at 1 μM of aminoindan. Thus, a concentration dependent attenuation of AMPA receptor induced increases of population spike amplitudes was recognized for rasagiline and even more for its metabolite aminoindan. On the opposite, virtually no effect could be seen in the presence of selegiline up to a concentration of 5 μM. Thus, a clear difference could be observed between rasagiline and aminoindan on one site and selegiline on the other side with respect to functional antagonism also of AMPA glutamate receptor stimulation.

### d) Functional interference with Kainate receptor activation

In order to test a possible interference of rasagiline, aminoindane or selegiline with Kainate receptor activation, glutamatergic neurotransmission was stimulated by ATPA, a very potent and selective Kainate receptor agonist. A concentration of 50 nM induced a significant enhancement of the populations spike amplitude. Under the condition of single stimuli increase of the amplitude from 1097 to 1904 μV (174% of control value) could be observed (Table 2). Virtually no effect on this signal could be seen in the presence of rasagiline or aminoindan up to a concentration of 5 μM. However, in the presence of selegiline the amplitude remained at control values (changing from 1083 to 1257 μV). Statistically significant differences to the ATPA induced increase were observed already with 2.5 μM of selegiline (p < 0.01). Similar results were obtained in the presence of theta burst stimulation. ATPA increased the amplitude to 3055 μV. Selegiline at a concentration of 5 μV attenuated the ATPA-induced signal down to 2134 μV. Thus, a concentration dependent attenuation of Kainate receptor induced increases of population spike amplitudes was recognized only for selegiline but not for rasagiline or aminoindan. Again a clear difference could be observed between the effects of rasagiline and aminoindan on one site and selegiline on the other side, but in a reversed manner.

**Table 2:** Amplitudes of population spike

	Single Stimulus	Theta Burst Stimulus
	μV	μV
**RS-ATPA 0.05 μM**	-1904.2 ± 55.4	-3054.5 ± 42.2
**+ Rasagiline 5.00 μM**	-1789.0 ± 54.6 n.s	-2998.5 ± 108.3 n.s
**+ Aminoindan 5.00 μM**	-1946.1 ± 58.8 n.s	-2850.8 ± 92.1 n.s
**+ Selegiline 2.50 μM**	-1616.1 ± 37.8 p < 0.01	-2531.3 ± 136.4 p < 0.01
**+ Selegiline 5.00 μM**	-1256.7 ± 53.2 p < 0.01	-2133.6 ± 48.4 p < 0.01

Effect of selegiline on Kainate receptor dependent increase of population spike amplitude, but lack of effect by rasagiline or aminoindan. Values are given as mean of n = 4 slices +- S.E.M. Statistical significance to ATPA signal is given as p-value.

### e) Functional interference with metabotropic glutamate receptor activation

**Figure 6:**
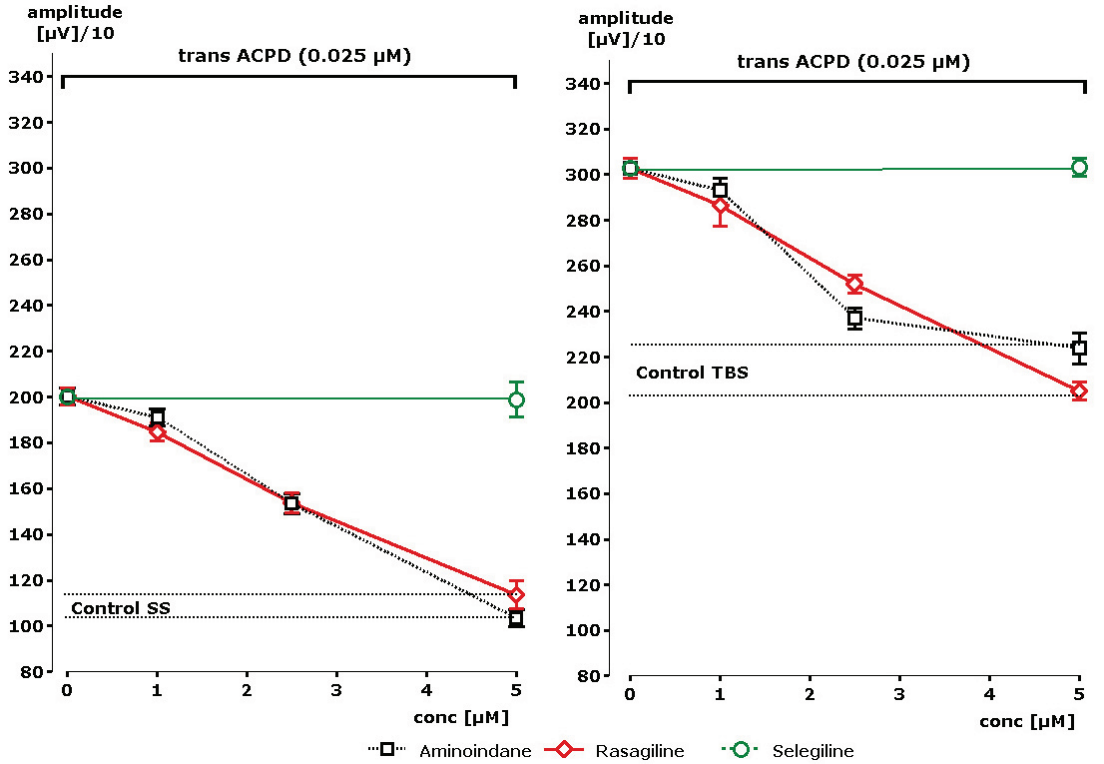
**Concentration dependent effect of rasagiline, aminoindan or selegiline in the presence of single stimuli (SS) or theta burst stimuli (TBS) after stimulation of the metabotropic glutamate receptor by ACPD**. Data are presented for the mean of n = 4 slices +- SEM. Statistically significant attenuation of pop-spike amplitude in comparison to ACPD-induced increases were obtained in the presence of 2.5 μM of rasagiline and aminoindan following single stimuli (SS) or theta burst stimulation. No effect was observed with selegiline. Stars indicate statistical significance of p < 0.05 in comparison to control.

In order to test a possible interference of rasagiline, aminoindan or selegiline with metabotropic glutamate receptor activation, ACPD, a very potent and selective metabotropic glutamate receptor agonist, was used to enhance pyramidal cell responses. A concentration of 25 nM induced a significant enhancement of the population spike amplitude. Under the condition of single stimuli increase of the amplitude from 1068 to 2003 μV (188% of control value) was observed (Figure 6). In the presence of rasagiline and SS conditions the amplitude remained at control value (changing from 1111 to 1134 μV). Statistically significant differences to the ACPD induced increase were observed with 1 μM of rasagiline (p < 0.01). Similar results were obtained in the presence of theta burst stimulation. ACPD increased the amplitude to 3027 μV. Rasagiline at a concentration of 5 μV attenuated the ACBD-induced signal down to control value (2050 μV). Thus, a concentration dependent attenuation of the metabotropic glutamate receptor induced increases of population spike amplitudes was recognized. Nearly identical results were seen in the presence of aminoindan (s. Figure 6). On the opposite, virtually no effect could be seen in the presence of selegiline with a concentration of 5 μM. Thus, a clear difference could be observed between rasagiline and aminoindan on one site and selegiline on the other side with respect to functional antagonism of metabotropic glutamate receptor stimulation.

## Discussion

The rat hippocampal in vitro slice preparation has been used under physiological and pathophysiological conditions. Two monoamine oxidase B inhibitors (rasagiline and selegiline) and one compound lacking monoamine oxidase B inhibition (aminoindan) have been compared with respect to their ability to attenuate glutamatergic transmission represented by decreasing responses of pyramidal cells to electric stimulation. This result is interpreted to represent functional neuroprotection against massive glutamatergic excitation.

Since simulation of ischemic conditions by oxygen-glucose deprivation (OGD) likewise resulted in showing that rasagiline and aminoindan prevented the breakdown of excitability, these effects probably also relate to neuroprotection (for selegiline this could be shown only to a minor degree). The term neuroprotection usually is taken to describe effects of drugs which might result in disease modifying actions during the course of Alzheimer's or Parkinson's illness. With respect to the latter, better neuroprotective and neurorestorative actions have been described for rasagiline in comparison to selegiline against lactacystin-induced nigrostriatal dopaminergic degeneration [27]. Also in a tissue culture model using PC12 cells under oxygen-glucose deprivation, rasagiline was clearly more effective than selegiline [28]. In addition, these authors could show that the neuroprotective effects of selegiline were blocked by its metabolite l-methamphetamine whereas aminoindan added to the effects of rasagiline. Taken together, all these findings suggest that the aminoindan moiety might be more important for neuroprotection than the propargyl moiety as suspected earlier [29]. Our results are therefore in line with earlier preclinical evidence for a neuroprotective action of rasagiline and its metabolite aminoindan. The functional impairment of glutamate dependent transmission obviously is not dependent on inhibition of monoamine oxidase B. However, a link between indirect inhibition of monoamine oxidase B and blockade of glyceraldehyde-3-phosphate dehydrogenase has recently been reported, which could also serve as an explanation for neuroprotective effects of rasagiline, selegiline and aminoindan [30].

The second part of the present investigation provides solid evidence that both rasagiline and selegiline interact functionally with glutamatergic receptor mediated transmission in addition to their known effects on MAO B, but by a different mechanism of action. The effects must be independent of the enzyme inhibition for the following reasons: firstly, aminoindan does not inhibit MAO B; secondly, both MAO inhibitors-rasagiline and selegiline-develop different receptor-mediated functional consequences within the glutamatergic system. This implicates that rasagiline and its metabolite aminoindan probably develop clinical properties different from that of selegiline.

A hypothesis exists that particular glutamate receptors of the N-methyl-D-aspartate type are over-activated in a tonic rather than a phasic manner, which under chronic conditions leads to neuronal damage [31]. Another clinical implication could be suspected from the combined attenuation of NMDA and AMPA receptor dependent effects: simultaneous administration of sub-threshold dosages of NMDA and AMPA antagonists had a positive influence on the development of L-dopa induced dyskinesias in rats and monkeys [32]. These data are corroborated by earlier findings showing glutamate super sensitivity in the putamen of Parkinson patients treated chronically with L-dopa [33]. A common disadvantage of currently available rather unselective NMDA receptor antagonists is the occurrence of adverse effects like hallucinations [34]. Therefore, rasagiline and its metabolite aminoindan, which do not induce such side effects, but not selegiline with methamphetamine as its metabolite, should have a positive effect on motor fluctuations in Parkinson patients.

With respect to the involvement of metabotropic glutamate receptors in Parkinson's disease there is evidence that they are involved in the pathologically altered circuitry in the basal ganglia. Several antagonists at this receptor alleviated L-dopa induced dyskinesia in 6-OH DA-lesioned rats [35]. Spontaneous firing of neurons in primate pallidum was increased by metabotropic glutamate receptor agonist DHPG and decreased by selective antagonists [36], which is in line with our results. Since glutamatergic input from the subthalamic nucleus shows over-activity during the disease, antagonists very well could compensate for this.

## Conclusions

Taking the effects of rasagiline and aminoindan together, not only neuroprotective effects could be measured but attenuation of NMDA, AMPA and metabotropic receptor mediated over-excitability of the glutamatergic system, also motor complications in Parkinson's disease-induced by imbalance of the glutamatergic system-should be ameliorated by a monotherapy with rasagiline. In addition, the newly discovered mechanism of action of rasagiline and aminoindan should be considered in the light of an extension of the clinical indication i.e. to treat Alzheimer's disease (for relation between Alzheimer's disease and glutamatergic system [37,38]. Last not least, over-activation of the glutamatergic system also is one of the consequences during stroke, amyotropic lateral sclerosis, Huntington's disease and neuropathic pain [39]. It remains to be tested if pharmacological intervention by rasagiline and its metabolite aminoindan provides a valuable therapeutic strategy for treatment of these diseases in addition to treatment of Parkinson's disease.

## Authors' contributions

WD provided the electrophysiological technology, supervised the performance of the experiments, gave interpretation of the results and wrote the manuscript. JAH initiated the study and made major contributions to the design. He also provided important information on the pharmacology of the preparation. All authors read and approved the final manuscript.
